# A Retrospective Analysis of Non-Attendance (DNA) in Haringey CAMHS: Addressing Barriers to Care and Improving Engagement

**DOI:** 10.1192/bjo.2025.10670

**Published:** 2025-06-20

**Authors:** Rakesh Sharma

**Affiliations:** CAMHS St Ann’s Hospital, London, United Kingdom

## Abstract

**Aims:** To evaluate DNA rates and contributory factors within CAMHS to propose interventions that improve appointment adherence and service delivery.

**Methods:** A retrospective review of appointment data and patient records.

Data sources:

RiO (electronic patient records).

Appointment scheduling logs.

Communication records (e.g. letters, SMS).

Audit Period: October 2024–December 2024.

Inclusion criteria:

All scheduled CAMHS appointments during the audit period.

Patients aged [5–18 years].

Exclusion criteria:

Appointments cancelled in advance by patients or clinicians.

Patients discharged prior to the scheduled appointment date.

**Results:** The high DNA rates, especially in the Generic Team (77–97 across three months), underline the wasted resources and delayed care. This validates the need to identify and address the root causes of DNAs.

Findings:

Generic team has consistently higher DNA rates.

ADHD and Adolescent Outreach teams also show engagement challenges.

**Conclusion:** The audit highlights significant DNA challenges in CAMHS. Addressing these issues through improved communication, flexible scheduling, and robust follow-ups can enhance patient engagement and resource efficiency. Future re-audits will track improvements and refine interventions.

